# Circular RNA circ0005276 promotes the proliferation and migration of prostate cancer cells by interacting with FUS to transcriptionally activate XIAP

**DOI:** 10.1038/s41419-019-2028-9

**Published:** 2019-10-17

**Authors:** Yang Feng, Yuxuan Yang, Xiaodan Zhao, Yameng Fan, Long Zhou, Jie Rong, Yan Yu

**Affiliations:** 1 School of Public Health, Xi’an Jiaotong University Health Science Center, Xi’an, China; 2 Xi’an Jiaotong University Health Science Center, 710061 Xi’an, China

**Keywords:** Cancer, Cell biology

## Abstract

Prostate cancer (PCa) is one of the major men’s malignancies with high mortality worldwide. Circular RNAs (circRNAs) have been shown to serve as essential regulators in human cancers. CircRNA can exert their functions by cooperating with their host genes. In the present study, microarray analysis revealed an upregulated mRNA in PCa samples. X-linked inhibitor of apoptosis protein (XIAP), a key regulator in the progression of human cancers. Through bioinformatics analysis, we determined that XIAP is a host gene for circRNA0005276. Therefore, this study focused on the interaction between circ0005276 and XIAP as well as their functions in PCa progression. The upregulation of XIAP and circ0005276 was determined in PCa tissues and cell lines. Moreover, we confirmed the positive regulation of circ0005276 on XIAP expression. Functionally, we validated that circ0005276 and XIAP promoted cell proliferation, migration and epithelial–mesenchymal transition. Mechanistically, we verified that circ0005276 interacted with FUS binding protein (FUS) so as to activate the transcription of XIAP. Rescue assays were conducted to determine the crucial role of XIAP in circ0005276 and FUS-mediated PCa cellular processes. Collectively, our study revealed the mechanism and function of circ0005276 and its host gene XIAP in PCa progression.

## Introduction

Prostate cancer (PCa), a prevalent human malignancy whose 5-year survival is merely around 29%^1^. The percentage of PCa in all cancer related death is about 13%^2^. Despite the development of comprehensive treatment, the prognosis of patients with PCa is still unfavorable due to the recurrence and metastasis^3^. Hence, novel potential therapeutic methods for PCa patients are exigently needed.

As a distinct group of noncoding transcripts, circular RNAs (circRNAs), form a closed continuous loop with the 3′RNA and 5′ RNA joined covalently^4–6^. In the past 40 years, circRNAs have been identified in eukaryotic cells by electron microscopy^7,8^ and were previously considered as splicing error by-products. Through the application of high-throughput sequencing and bioinformatics, circRNAs have been successively identified in multiple cell lines and various species^9–11^. Most circRNAs are formed by exon or intron back-splicing. This process differs from the formation of linear RNAs. Two mechanisms exist for the formation of exonic or exon–intron circRNAs: exon skipping and back-splicing^12,13^. Previous studies reported circRNAs as “miRNA sponges” that play an inhibitory role in miRNA regulation^14,15^. Known as “miR-7 sponges,” antisense to the cerebellar degeneration-related protein 1 (CDR1as), also known as ciRS-7, is one of the most widely known and effective circRNAs^16,17^. Recent years, reports associated with circRNAs have been published to elucidate the correlation between circRNAs and cancer^18–20^. Therefore, circRNAs may be critical biological markers in the identification of disease mechanisms and for developing new methods for precise diagnosis and effective treatment of human cancers. CircRNAs stem from their host genes and may have regulatory relationship with their host genes. In the present study, top 500 mRNAs that were significantly upregulated in PCa tissues were screened out. Among which, X-linked inhibitor of apoptosis protein (XIAP) exhibited the highest fold change. According to previous reports, XIAP is closely related to tumorigenesis and development by acting as an oncogene in cancers^21–24^. Therefore, we chose it to be a research object. According to bioinformatics analysis, XIAP is the host gene of circ0005276. To analyze their potential role, we examined the expression level of XIAP and circ0005276 in 90 pairs of PCa and adjacent normal samples. Both upregulation of XIAP and circ0005276 was determined in PCa tissues and cell lines. Moreover, circ0005276 had positive effect on the expression of pre-mature and mature XIAP mRNA.

circRNAs play crucial roles in regulating gene expression at transcriptional level by interacting with RNA binding proteins^25–27^. In the present study, RNA binding protein FUS was found to be interacted with circ0005276 in PCa. Interestingly, circ0005276 and FUS were explored as the regulators for the transcription of XIAP. Finally, rescue assays were conducted to demonstrate the role of XIAP in circ0005276 or FUS-mediated PCa progression. Collectively, this study revealed the novel mechanism of circ0005276 in PCa progression.

## Materials and methods

### Patient samples and cell culture

Fresh PCa tissues and pair-matched adjacent normal tissues were collected from 90 patients from June 2012 and July 2017 at Xi’an Jiaotong University Health Science Center. PCa tissues and normal tissues from surgery were snap-frozen in liquid nitrogen until use. Patients who had received preoperative treatment were excluded from the study. Our study had acquired the approval of the Research Ethics Committee of the hospital and the written informed consents from all participants.

One human normal prostate epithelial cell line (RWPE-1) and four PCa cell lines (PC-3, DU145, VCaP and LNCaP) were acquired commercially from American Type Culture Collection (ATCC, Manassas, VA, USA). PCa cell lines were allowed to grow in RPMI 1640 medium (Gibco BRL, Grand Island, NY, USA) containing 10% fetal bovine serum (FBS). RWPE-1 was cultured in DMEM (Gibco BRL) containing 10% FBS. Culture plates were kept in a humidified incubator with 5% CO_2_ at 37 °C.

### RNA isolation and qRT-PCR

Total RNA was first extracted from cell lines or tissues by using TRIzol (Invitrogen, Carlsbad, CA, USA) based on the guidebook. Total RNA was transcribed reversely into cDNA using M-MLV Reverse Transcriptase (Invitrogen). Real-time PCR was performed with SYBR Prime Script PLUS RT-RNA PCR Kit (Takara, Tokyo, Japan). The primers were listed as follows: XIAP 5′-ACTTCGGGTTTCACGACTCC-3′ (forward) and 5′-TACACAAGCTGTACCCACCG-3′ (reverse); circ0005276 divergent: GCTAAATGGTATCCAGGGTGC (forward) CCCTCCTCCACAGTGAAAGC (reverse); FUS 5′-GGTACTCAGCGGTGTTGGAA-3′ (forward) and 5′-CGCCCTTACCTACCGTTTGA-3′ (reverse); GAPDH 5′-GCTTTCTTTCCTTTCGCGCT-3′ (forward) and 5′-TTTGCGGTGGAAATGTCCTT-3′ (reverse). The relative gene expression levels were analyzed by 2^−∆∆Ct^ with the normalization to GAPDH.

### Cell transfection

PC-3 and DU145 cell lines were planted in six-well plates until cell confluence reached ~70%. The short hairpin RNAs (shRNAs) specific to XIAP, circ0005276 and FUS (sh-XIAP#1/2/3, sh-cric#1/2/3 and sh-FUS#1/2/3, Invitrogen, Carlsbad, CA, USA) were synthesized and transfected into cell lines using Lipofectamine2000 (Invitrogen) following the standard method. It is known that circ0005276 has overlapped sequence with its host gene XIAP, we designed the shRNA sequence for circ0005276 in its back-spliced junction region. shRNA sequences for circ0005276 are shown as follows: sh-circ#1: CCGGGAAGAATTGGTGAAGGTGATACTCGAGTATCACCTTCACCAATTCTTCTTTTTG; sh-circ#2: CCGGGAATTGGTGAAGGTGATAACTCGAGTTATCACCTTCACCAATTCTTTTTG; sh-circ#3: CCGGGCTAGAAGAATTGGTGAAGGTGCTCGAGCACCTTCACCAATTCTTCTAGCTTTTTG. Cells treated with sh-NC were seen as the control. To overexpress XIAP and FUS, cell lines were transfected with pcDNA-XIAP and pcDNA-FUS (termed XIAP and FUS) or the empty pcDNA3.1 vectors. At 48 h post transfection, cells were reaped for further study.

### Cell proliferation assays

In all, 1.0 × 10^3^/well transfected PC-3 and DU145 cells were plated in 96-well plates for 24 h. Ten microliter of CCK-8 solution (Dojindo Laboratories, Kumamoto, Japan) was added to each well. Followed by incubation at 37 °C for 2 h, cell viability was determined by assessing the absorbance at 450 nm at indicated time points (1, 2, 3, and 4 days). For EdU incorporation assay, the transfected cells were kept on sterile coverslips in 24-well culture plates. EdU kit (Invitrogen) was utilized to measure cell proliferation. Cells were imaged using Laser confocal microscopy (FV300, Olympus, Tokyo, Japan). Cell nucleus were exposed to 4′,6-diamidino-2-phenylindole (DAPI). Experimental data were shown as the percentage of EdU positive cells (green) to total DAPI positive cells (blue).

### Transwell assays

Cell invasive ability was evaluated with Matrigel-coated Transwell chamber (Corning Incorporated, Corning, NY, USA) in accordance with supplier’s protocol. Cell migration assay was analyzed using Transwell chamber without Matrigel. PC-3 and DU145 cells (5000) were seeded into the upper chamber with serum-free RPMI 1640. The complete medium was placed into the lower chamber. Cells invading or migrating through the membrane were fixed in 4% paraformaldehyde and dyed with crystal violet. Five random fields from each well were and counted and recorded using a microscope (magnification, ×200).

### Western blotting

Protein samples from tissues or cell lines were diluted in loading buffer to the same concentrations, followed by separation by electrophoresis on 10% SDS-PAGE. The total proteins were transferred on Hybond membrane (Amersham, Munich, Germany) and sealed in 5% skimmed milk at room temperature for 2 h. Afterwards, membranes were incubated with primary antibodies against E-cadherin (ab76055, 1:1000, Abcam, Cambridge, UK), N-cadherin (ab76057, 1:1000), XIAP (ab21278, 1:1000) and GAPDH (ab9485, 1:2500) all night at 4 °C. Followed by thrice washing in Tris-buffered saline (TBST), membranes were treated with relative secondary antibodies (1:2000) for 2 h. At length, enhanced chemiluminescence reagent (Santa Cruz Biotechnology, Santa Cruz, CA, USA) was applied to visualize protein bands.

### Immunofluorescence staining

PC-3 and DU145 cell lines were seeded on cover slips for one day and fixed in cold acetone for half an hour. After incubation with antibodies against E-cadherin and N-cadherin all night, cells were treated with goat anti-rabbit FITC conjugated secondary antibodies (Abcam) for 1 h. Thereafter, cells were rinsed extensively. Nuclei was stained with DAPI. The stained cells were observed using a fluorescence microscope (Olympus, Tokyo, Japan).

### Animal study

Male nude mice, aged ~6 weeks, were kept in an SPF-grade pathogen-free animal laboratory. This animal study had obtained the approval of the Animal Research Ethics Committee of Xi’an Jiaotong University Health Science Center. In all, 5 × 10^6^ PC-3 cells were transfected and subcutaneously injected around the left flank of the nude mice (five mice per group). Tumor volume was examined as 0.5 × length × width^2^ every 7 days. Four weeks later, mice were killed, tumor were excised and weighed for further study.

### Nuclear-cytoplasmic fractionation

In all, 5 × 10^6^ PC-3 and DU145 cells were rinsed twice in pre-chilled phosphate buffer saline (PBS) and suspended in cell fraction buffer. Followed by incubation on ice for 10 min, cells were centrifuged. The upper solution was removed. The nuclear pellet was collected to extract RNA in cell disruption buffer. At last, the isolated RNA was measured by qRT-PCR, normalizing to GAPDH (cytoplasmic control) and U6 (nuclear control).

### RNA pull-down assay

For RNA pull-down assay, RNAs were first labeled with biotin using Biotin RNA Labeling Mix (Roche Molecular Systems, Inc., Hague Road, IN), followed by in vitro transcription using T7 RNA polymerase (Roche). Biotinylated cells were purified and cultured with protein lysates. Subsequently, cells were subjected to streptavidin agarose beads (Life Technologies, Carlsbad, CA, USA) for an hour at room temperature. Afterwards, beads were eluted thrice in Biotin Elution Buffer and boiled in SDS buffer. The retrieved proteins were analyzed by qRT-PCR. IgG was considered as the control.

### Fluorescence in situ hybridization analysis

The Fluorescence in situ hybridization analysis (FISH) probe for circ0005276 was designed and synthesized by Biosearch Technologies, Inc. (Petaluma, CA, USA). PC-3 and DU145 cell lines in slides were cultured in 4% paraformaldehyde for 30 min and digested with protein K at 37 °C for 20 min. After washing in PBS, cells were subjected to dehydration with ascending series of ethanol. Thereafter, the denatured cells were incubated with 2 μL of FISH probe and 18 μL of hybridization reaction solution at 42 °C overnight. After hybridization, slides were rinsed three times in 2 × SSC and counterstained with DAPI. Cells were finally exposed to fluorescent signal detection using LSM800 confocal laser microscopy (ZEISS, Jena, Germany).

### Chromatin immunoprecipitation assay

For Chromatin immunoprecipitation (ChIP) assay, SimpleChIP^®^ Enzymatic Chromatin IP Kit was bought from Cell Signaling Technology (CST, Danvers, MA, USA). The crosslinked chromatin was digested with enzyme to break into 200–1000-bp fragments. Immunoprecipitation was performed using 2 μg of anti-XIAP (Abcam), 2 μg of anti-FUS (Abcam) and 2 μg of IgG with rotation all night at 4 °C. Then, 30 μl of magnetic beads were added into the reaction for 2 h at 4 °C. Immunoprecipitated chromatin was recovered and measured by qRT-PCR.

### DNA pull-down assay

According to previous report, DNA pull-down assay was carried out^28^. Briefly, DNA pull-down was conducted in indicated cells by using 300 μg nuclear extract which was supplemented with 5 μg of XIAP in a buffer containing some compounds, including 140 mM NaCl, 1.5 mM MgCl2, 20% Glycerol, 0.2 mM EDTA, 20 mM HEPES pH 7.6, 10% Triton X-100 that was supplemented with the protease inhibitor with a dilution of 1:1000. All these reagents and compounds were obtained from Sigma-Aldrich, St Louis. (MO, USA). Twenty microgram of non-biotinylated oligonucleotides were used to avoid nonspecific binding. Samples were incubated all night. After washing, western blot was applied to detect bound proteins.

### Luciferase reporter assay

To perform XIAP promoter analysis, PC-3 and DU145 cells were planted into 24-well culture plates. XIAP promoter was amplified by PCR and cloned into pGL3-Basic vector (Promega Corporation, Madison, WI, USA). Cells were co-transfected with the pcDNA-FUS vector, the Renilla plasmid (Promega) and the plasmids containing XIAP promoter. After 48 h, the relative luciferase activity was detected with the Dual-Luciferase Reporter Assay System (Promega).

### Microarray analysis

Total RNA from three PCa tissues and three matched adjacent normal tissues was purified using the RNeasy Mini Kit (Qiagen, Valencia, CA, USA). Sample preparation and microarray hybridization were carried out in accordance with the user guide. The differentially expressed genes in cancerous tissues were screened out with the criteria of *P* < 0.05 and fold-change >2. In addition, the total RNA isolated from cells transfected with sh-FUS or sh-XIAP along with their corresponding controls (sh-NC) were subjected to microarray processing.

### Bioinformatics analysis

The host gene of circ0005276 was determined by searching from UCSC (http://genome.ucsc.edu/). The DNA motif of FUS and the binding sequence between FUS and XIAP promoter were obtained by using the online tool JASPAR (http://jaspar.genereg.net/).

### Statistical analysis

Data of all experiments were expressed as mean ± standard deviation (SD). Each experimental procedure was conducted at least in triplicate. GraphPad Prism version 5.0 (GraphPad Software, La Jolla, CA, USA) was applied to analyze data. Group comparisons were analyzed using Student’s t-test or one-way ANOVA with the significant level of p value less than 0.05. The relevance between the expressions was analyzed via Pearson’s correlation analysis.

## Results

### Circ0005276 and XIAP were upregulated in prostate cancer

**Fig. 1:**
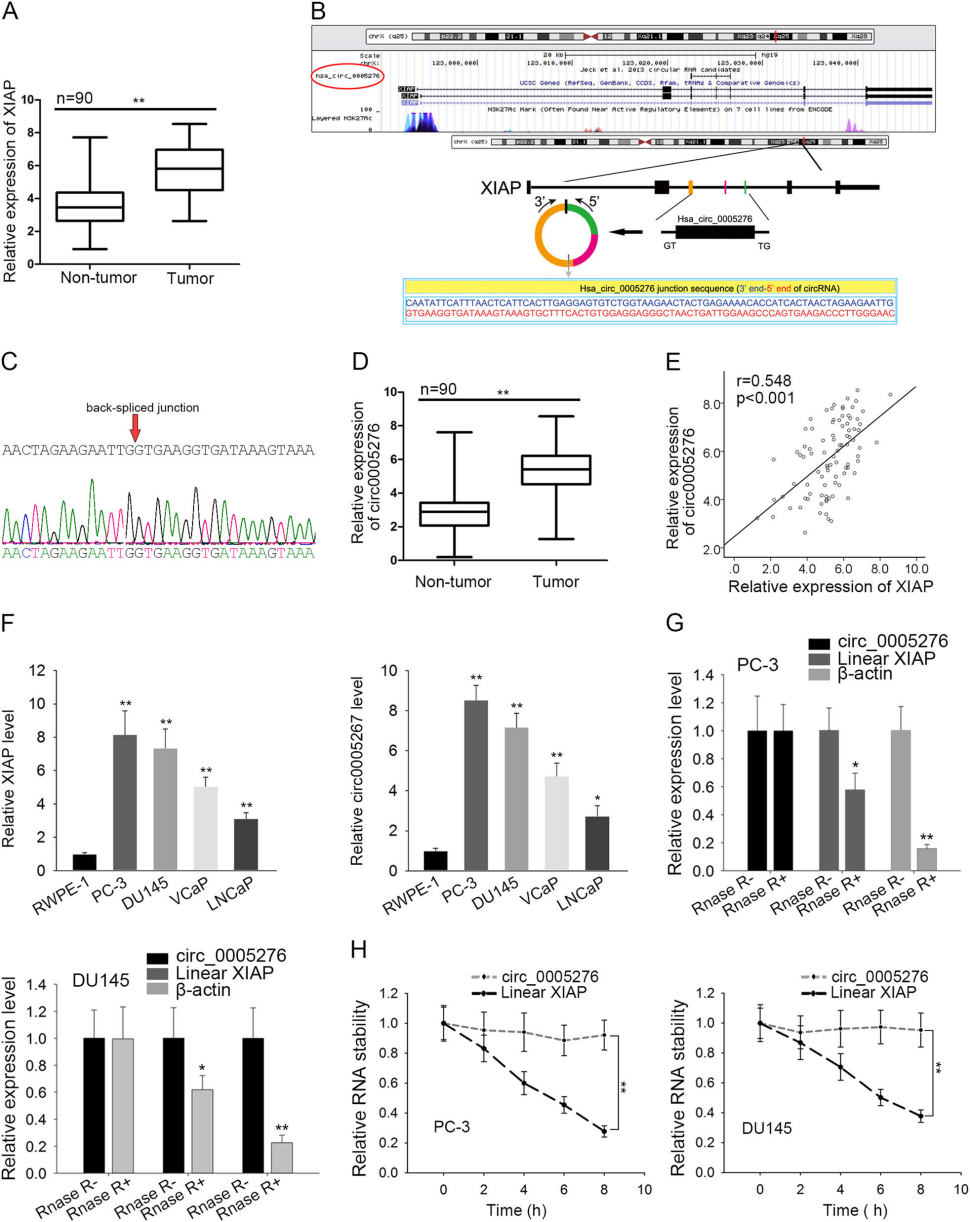
Circ0005276 and XIAP were upregulated in prostate cancer. **a** mRNA level of XIAP in PCa tissues and paired normal tissues. **b** XIAP was identified as the host gene for circ0005276 as obtained from UCSC. **c** The back-spliced junction of circ0005276. **d** Relative expression level of circ0005276 in PCa tissues. **e** The expression association between XIAP and circ0005276 in PCa tissues. **f** The expression level of XIAP and circ0005276 in four PCa cell lines and one prostate epithelial cell line. **g** RNA expression level in cells treated with or without Rnase R. **h** Stability of circ0005276 and XIAP in cells treated with actinomycin D. ^*^P < 0.05, ^**^P < 0.01, ^**^P < 0.001

First of all, microarray analysis was applied to analyze the differentially expressed mRNAs in PCa tissues. Among which, XIAP was the mRNA which exhibited the highest fold change (Supplementary Fig. 1A). All these 500 upregulated mRNAs were subjected to GO and KEGG pathway analysis. As illustrated in Supplementary Fig. 1B, C, upregulation of these mRNAs might be correlated with cell proliferation, migration or transcriptional regulation. Upregulation of XIAP was further validated in 90 PCa tissues compared to adjacent normal controls (Fig. 1a). Searching from UCSC, we analyzed that the XIAP is the host gene of circ0005276 (Fig. 1b). Then, circ0005276 was subjected to circular RNA sequencing (Supplementary Fig. 2). The back-spliced junction of circ0005276 was determined by sequencing technology (Fig. 1c). Similarly, circ0005276 showed higher expression level in PCa tissues (Fig. 1d), which was consistent with XIAP (Fig. 1e). Additionally, four commonly used PCa cell lines (PC-3, DU145, VCaP, and LNCaP) and one normal prostate epithelial cell line (RWPE-1) were collected to detect the expression of XIAP and circ0005276. Relative high level of XIAP or circ0005276 was determined in four PCa cell lines (Fig. 1f). Both of them were expressed highest in PC-3 and DU145 cell lines. We chose these two cell lines for all subsequent experiments. To further validate the circular characteristics of circ0005276, we treated PCa cells with Rnase R and actinomycin D. It was uncovered that treatment with Rnase R and actinomycin D had no significant effect on the stability of circ0005276 but had obvious effect on the stability of linear XIAP (Fig. 1g, h). These data suggested that circ0005276 and it host gene XIAP might be regulators in PCa.

### The potential regulatory relationship between circ0005276 and XIAP

**Fig. 2:**
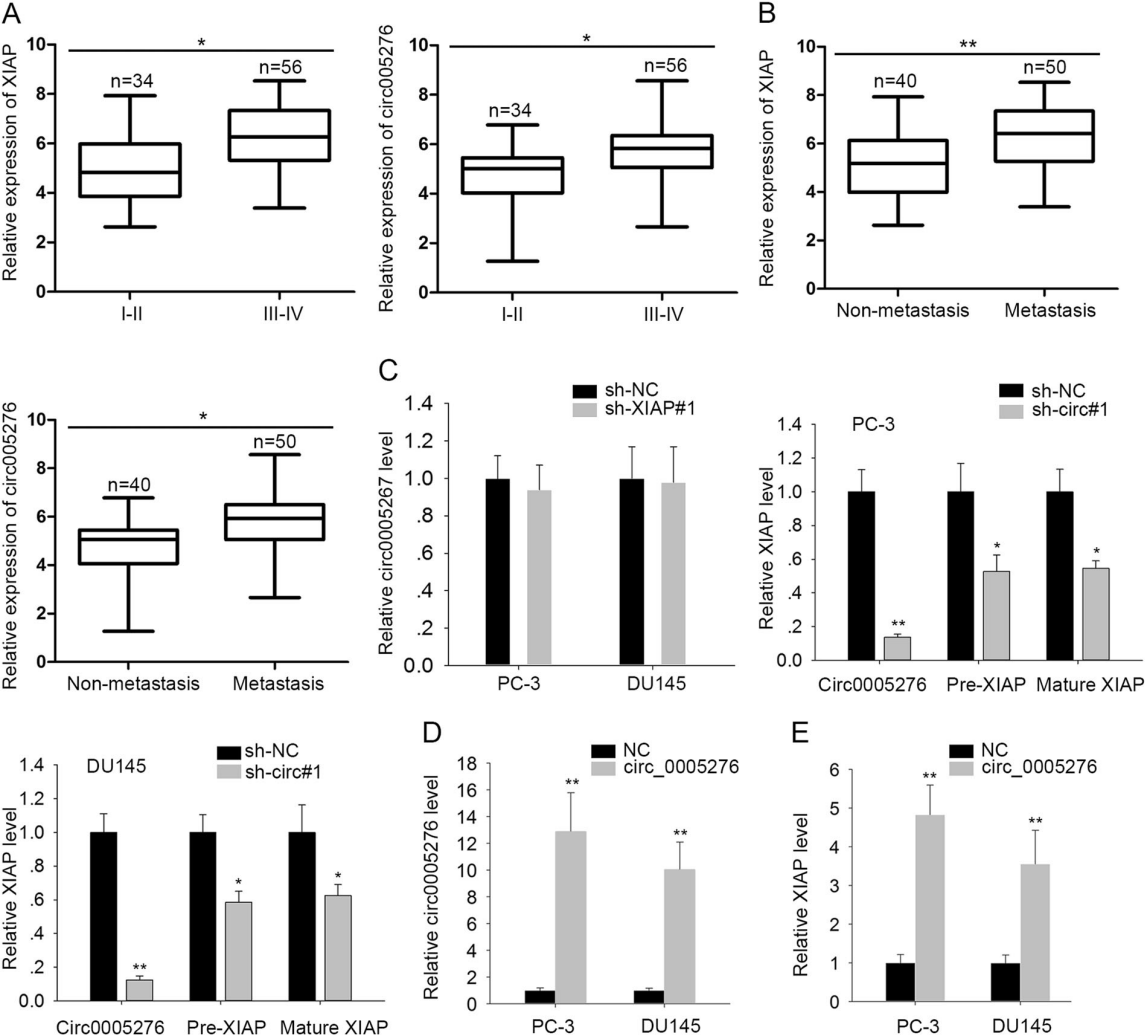
The potential regulatory relationship between circ0005276 and XIAP. **a**–**b** The expression level of XIAP and circ0005276 in PCa tissues with different tumor stages and metastatic conditions. **c** The effect of XIAP knockdown on the circ0005276 expression or the effect of circ0005276 knockdown on the XIAP expression. **d**–**e**Overexpression efficiency for circ0005276 and its effect on XIAP expression. ^*^P < 0.05, ^**^P < 0.01

Further, the expression of XIAP and circ0005276 was detected in tumor tissues with different tumor stage and metastatic condition. It was observed that XIAP and circ0005276 were both expressed higher in tissues with advanced stage and metastasis (Fig. 2a, b). The knockdown efficiency for XIAP or circ0005276 was measured and determined in PC-3 and DU145 cell lines (Supplementary Fig. 3A, B). The highest knockdown efficiency was observed in cells transfected with sh-XIAP#1 or sh-circ#1. Therefore, we chose these two plasmids for all subsequent experiments. In addition, we found that knockdown of XIAP had no obvious impact on circ0005276 expression (Fig. 2c), while silencing of circ0005276 decreased the level of XIAP in two PCa cell lines. Additionally, overexpression of circ0005276 (Fig. 2d) enhanced the level of XIAP in PCa cell (Fig. 2e). These results indicated a potential regulatory effect of circ0005276 on the XIAP expression.

### Knockdown of XIAP or circ0005276 suppressed PCa cell growth and migration

**Fig. 3:**
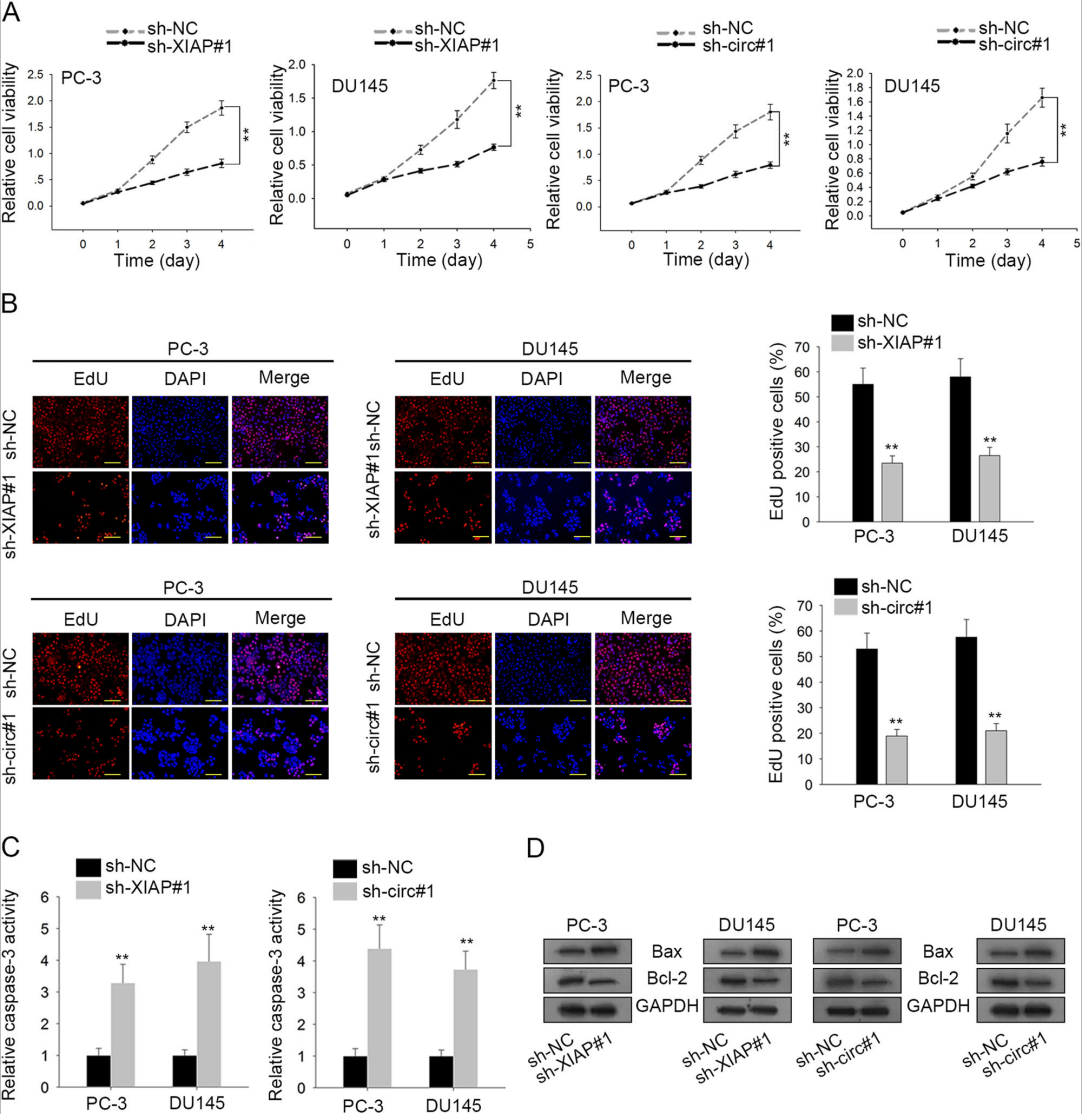
Knockdown of XIAP or circ0005276 inhibited cell growth. **a–b** Cell viability or proliferation in PC-3 and DU145 cells transfected with sh-XIAP#1 or sh-circ#1 by CCK-8 and EdU assay (Scale bar = 200 μm). **c** Caspase-3 activity was tested in PCa cells after circ0005276 or XIAP was downregulated. **d** Apoptosis-related proteins (Bax and Bcl-2) were detected in cells after transfection. ^**^P < 0.01

**Fig. 4:**
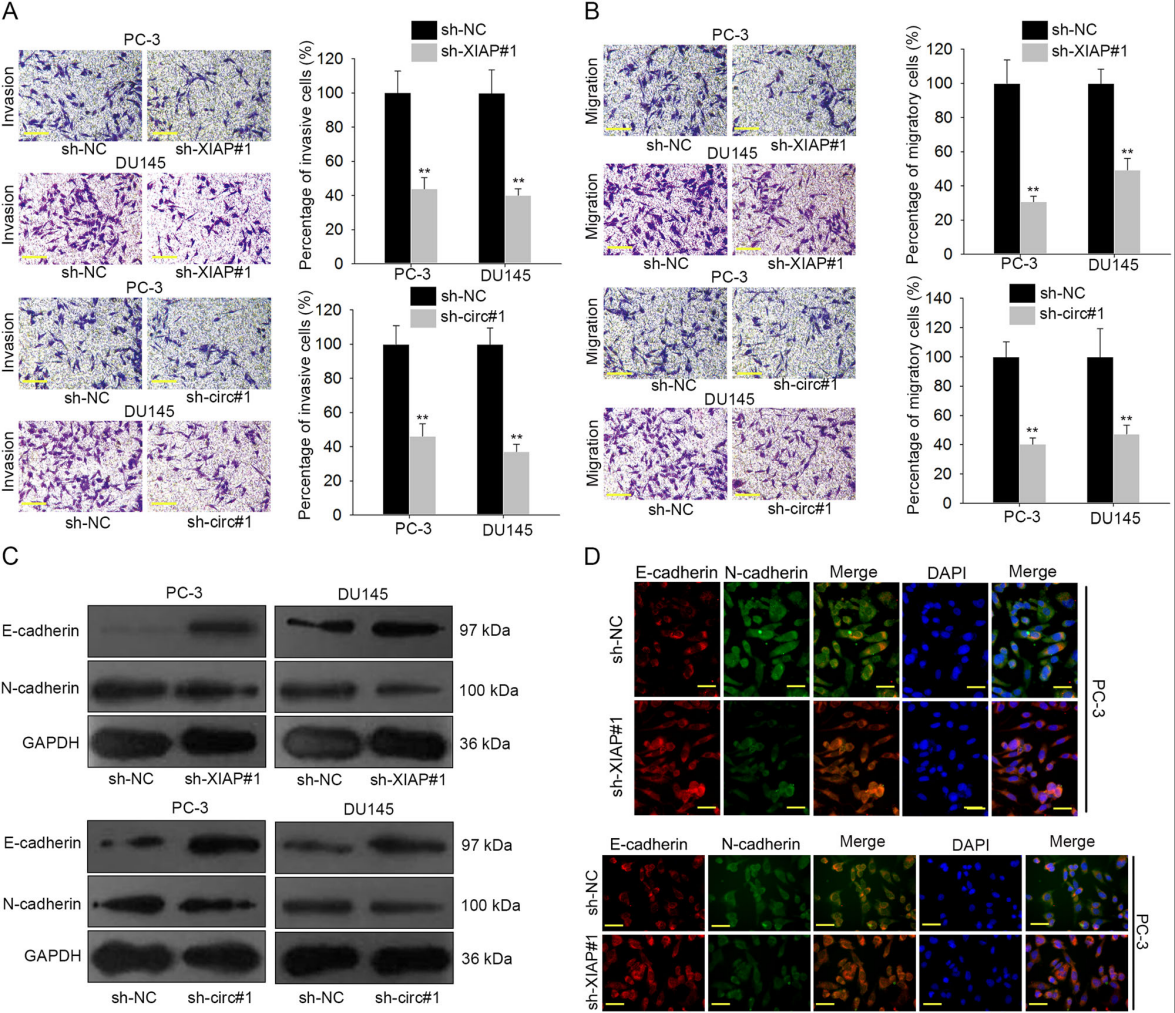
Knockdown of XIAP or circ0005276 suppressed PCa cell invasion, migration and EMT process. **a–b** Cell invasion or migration in PC-3 and DU145 cells transfected with sh-XIAP#1 or sh-circ#1. Scale bar = 200 μm. **c–d** The expression levels of E-cadherin and N-cadherin in XIAP- or circ0005276-downregulated PC-3 and DU145 cells. Scale bar = 200 μm.^**^P < 0.01

Considering the dysregulation of XIAP and circ0005276 in PCa tissues, we conducted loss-of function assays in above two cell lines. It was observed from CCK-8 and EdU assays, cell proliferation was efficiently suppressed by the knockdown of XIAP or circ0005276 (Fig. 3a, b). Moreover, we detected cell apoptosis in two PCa cells after silencing of circ0005276 or XIAP. It was uncovered that caspase-3 activity and Bcl-2 protein level were increased, while the protein level of Bax was reduced (Fig. 3c, d). Importantly, transwell assays revealed that both migration and invasion were suppressed with the inhibition of XIAP or circ0005276 (Fig. 4a, b). The change on the EMT process was observed in indicated PCa cell lines. The increased protein level of E-cadherin and the decreased level of N-cadherin reflected that EMT process might be suppressed by the downregulation of XIAP or circ0005276 (Fig. 4c and Supplementary Fig. 3C). The similar results were observed in indicated cells by immunofluorescence (Fig. 4d). In vivo experiments further validate that tumor growth was stagnated after silencing of circ0005276 or XIAP (Supplementary Fig. 3D–F). All results indicated that XIAP and circ0005276 acted as two oncogenes in PCa progression.

### Circ0005276 specifically interacted with FUS

**Fig. 5:**
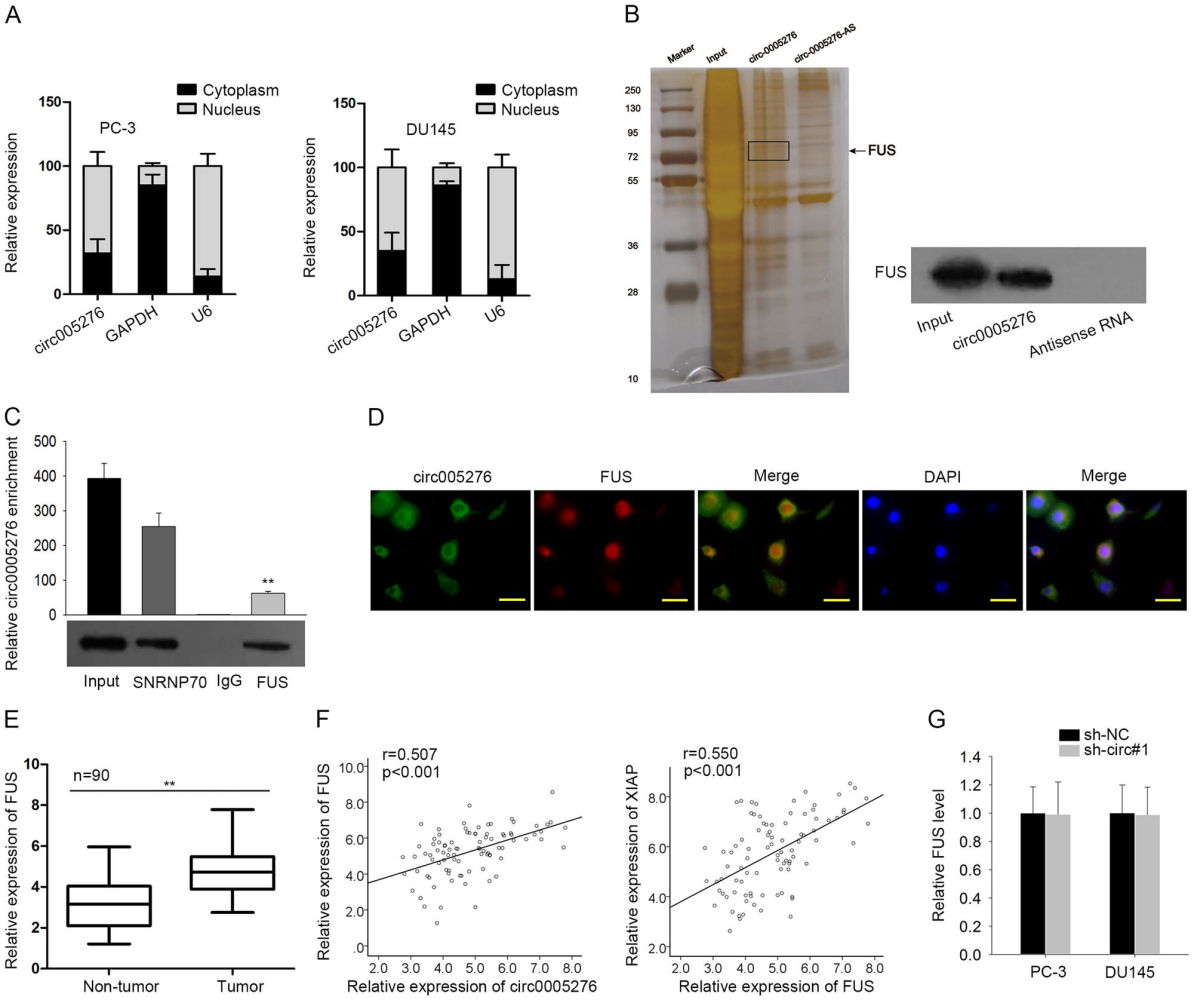
Circ0005276 specifically interacted with FUS. **a** The nuclear or cytoplasmic circ0005276 in PC-3 and DU145 cells. **b** Pull-down assay showed the interaction between circ0005276 and the RNA binding protein FUS. **c** The interaction between circ0005276 and FUS was determined by RIP assay. **d** The co-localization of circ0005276 and FUS in PCa cells. Scale bar = 200 μm. **e** The FUS expression in PCa and paired normal tissues. **f** The association between FUS expression and circ0005276 or XIAP expression in PCa tissues. **g** FUS expression in circ-0005276-downregulated PCa cells. ^**^P < 0.01, ^***^P < 0.001

Furtherly, we tunneled the mechanism through which circ0005276 exerted function in PCa progression. At first, localization of circ0005276 was determined in two PCa cell lines. According to the data illustrated in Fig. 5a, circ0005276 was located in both cytoplasm and nucleus. The significance of circRNA-protein interaction has been revealed during recent years, including with RNA binding proteins^29,30^. And a number of RBPs has been identified to have an interplay with circRNAs in cancers^31^, and can regulate gene expressions^26,27^. In this regard, we investigated whether circ0005276 can interact with a RBP to regulate its downstream genes. After mass spectrometry analysis and RNA pull-down assay, we determined that FUS was interacted with circ0005276 in PCa cell lines (Fig. 5b). The data for mass spectrometry analysis were shown in Supplementary Table 1. Their interaction was further demonstrated by RIP assay (Fig. 5c). Fused in sarcoma (FUS) is known to be an RBP whose interaction with circRNA has been uncovered in gastric cancer^31^. Also, we localized the expression of circ0005276 and FUS protein by FISH and Immunofluorescence. The overlapped localization of circ0005276 and FUS protein expression was identified in PCa cells (Fig. 5d). Furthermore, FUS was expressed at a relative high level in PCa tissues, which was consistent with that of FUS and XIAP (Fig. 5e, f). Furthermore, we detected the effect of silenced circ0005276 on FUS expression. There was no obvious effect of circ0005276 on FUS expression (Fig. 5g). Thus, we identified the interaction between circ0005276 and FUS protein.

### FUS exerted oncogenic function in PCa by cooperating with circ0005276 to regulate XIAP

**Fig. 6:**
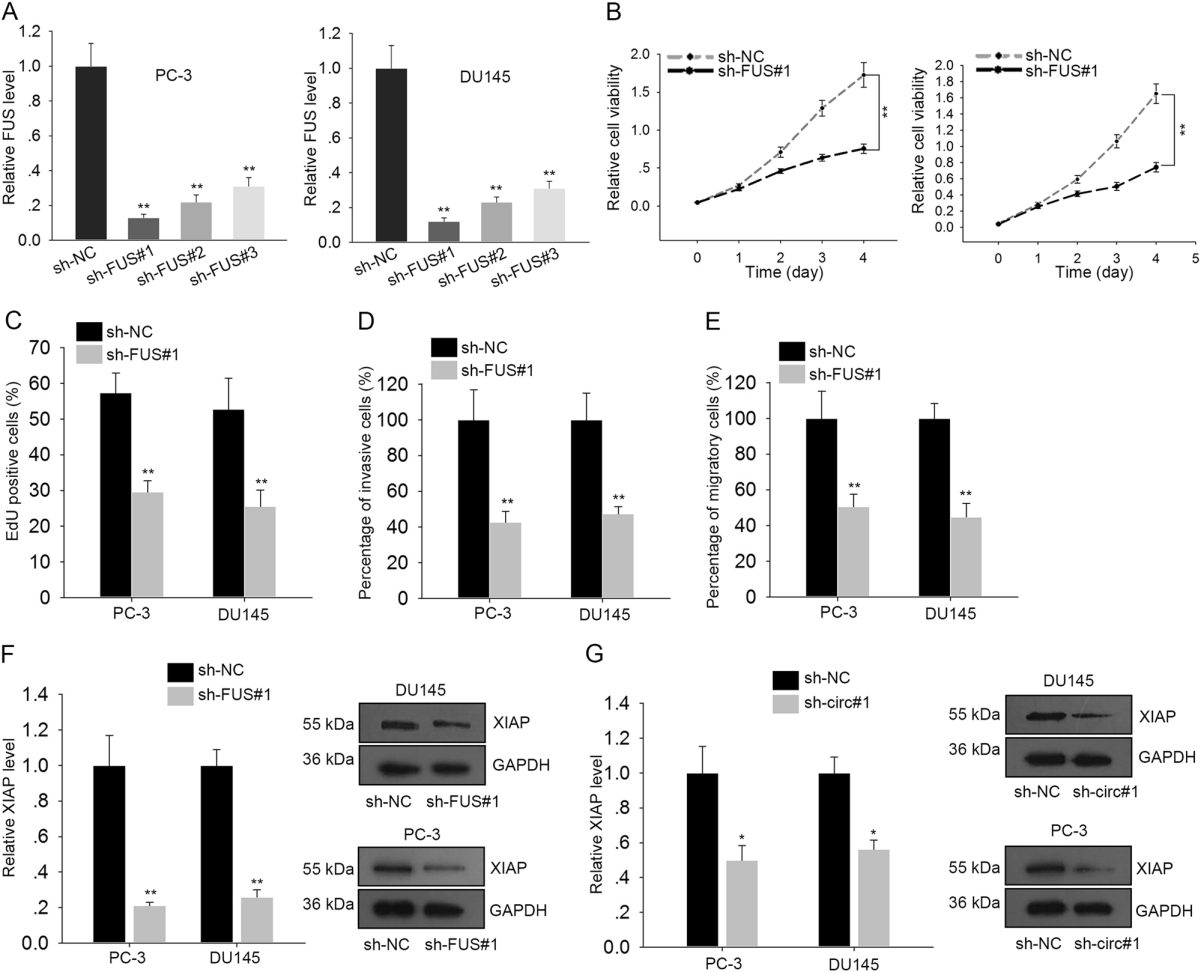
FUS exerted oncogenic function in PCa by cooperating with circ0005276 to regulate XIAP. **a** Transfection efficiency of specific shRNAs against FUS (sh-FUS#1/2/3) into PC-3 and DU145 cells. **b**–**c** Cell viability and proliferation in PC-3 and DU145 cells transfected with sh-FUS#1 or sh-NC. **d**–**e** Migratory or invasive ability of FUS-downregulated PC-3 and DU145 cells. **f**–**g** The mRNA and protein level of XIAP in cells transfected with sh-FUS#1 or sh-circ#1. ^*^P < 0.05, ^**^P < 0.01

Based on above, we knew that FUS was upregulated in PCa samples. Tp determine the role in PCa progression, loss-of function assays were carried out. At first, FUS was efficiently silenced in two PCa cell lines by three shRNAs, especially by sh-FUS#1 (Fig. 6a). Cell proliferation and transwell assays demonstrated that silencing of FUS led to the inhibition on cell proliferation, invasion and migration (Fig. 6b-e). What’s more, it has been reported to be a multifunctional RBP which can regulate transcription, RNA splicing, and mRNA stability^32,33^. Considering the positive expression association between FUS and circ0005276 or XIAP, we further analyzed whether circ0005276 positively regulate XIAP by recruiting FUS. qRT-PCR and western blot analysis showed that the mRNA and protein level of XIAP were decreased in cells transfected with sh-circ#1 or sh-FUS#1 (Fig. 6f, g and Supplementary Fig. 4A). These results suggested that FUS exerts oncogenic functions by binding with circ0005276 to regulate XIAP. To analyze whether FUS involved in circ0005276-mediated PCa cellular processes, rescue assays were conducted. According to the results illustrated in Supplementary Fig. 4B–D, overexpression of FUS had no significant effect on reversing sh-circ0005276-mediated cell proliferation and migration. Therefore, we confirmed that FUS was not the downstream target of circ0005276.

### Circ0005276 co-functioned with FUS to activate the transcription of XIAP

**Fig. 7:**
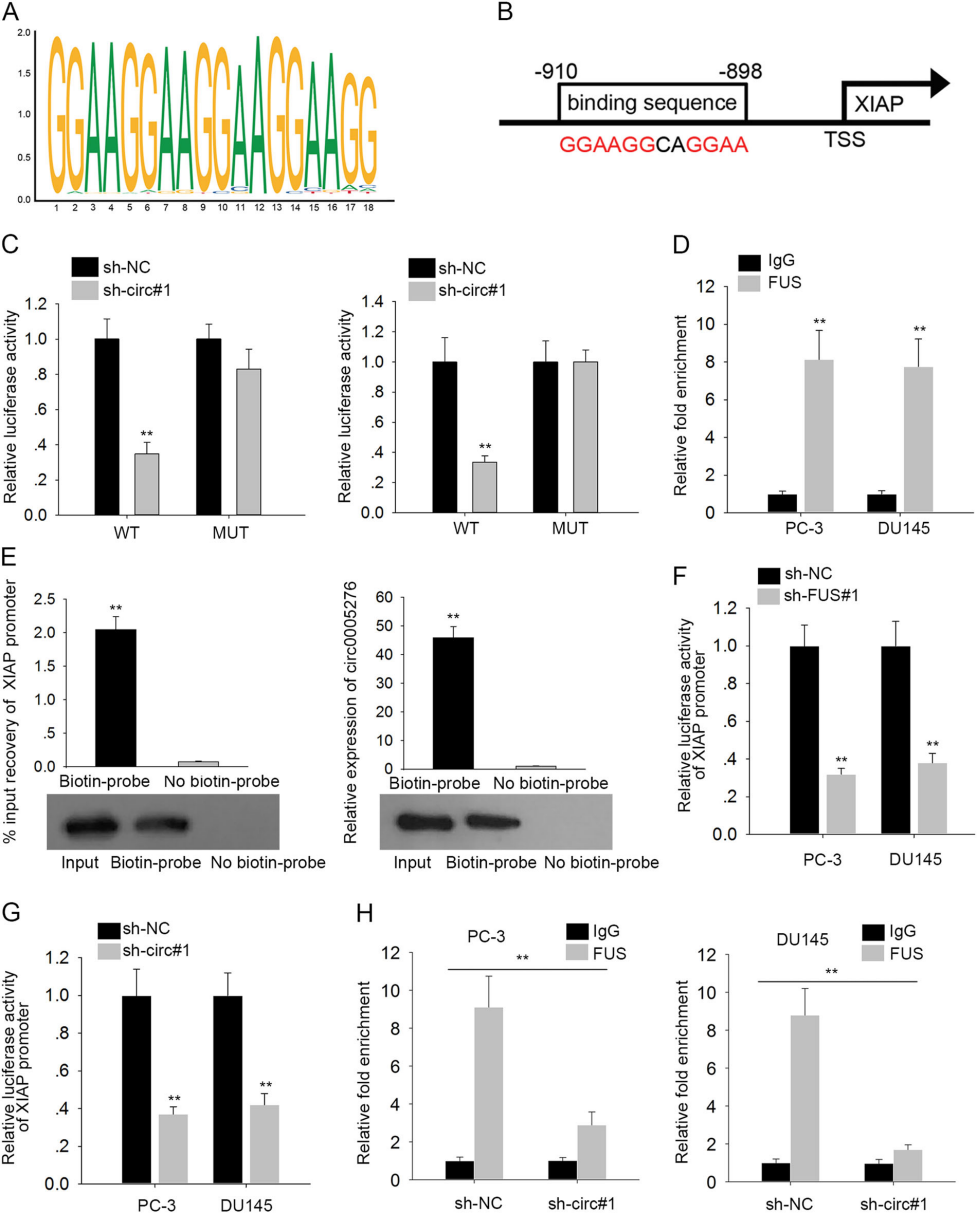
Circ0005276 co-functioned with FUS to activate the transcription of XIAP. **a** DNA motif of FUS obtained from JASPAR. **b** Putative binding sites between FUS and XIAP promoter. **c** The luciferase activity of reporters containing the putative binding sites or mutant binding sites was examined in response to circ0005276 knockdown. **d** ChIP followed by qPCR validated the interaction between FUS and XIAP promoter. **e** DNA pull-down assay revealed the binding of circ0005276/FUS complex to the XIAP promoter. **f**–**g** Luciferase reporter assay showed that silencing either circ0005276 or FUS weakened the luciferase activity of XIAP promoter. **h** ChIP assay was conducted to determine the effect of circ0005276 silence on the affinity of FUS to XIAP promoter. ^**^P < 0.01

It has been reported that FUS could regulate mRNA stability in cytoplasm, whereas regulate transcription in nucleus^32,33^. Since FUS was identified to be abundantly expressed in nucleus, we speculated that circ0005276 could regulate XIAP expression through FUS at transcription level. With the aid of bioinformatics tools, we compared the DNA motif of FUS with the promoter sequences of XIAP, founding that there existed binding sites between FUS and XIAP promoter (Fig. 7a, b). Moreover, we mutated the putative binding sites and found that the luciferase activity of mutant reporter was not affected by the knockdown of circ0005276 (Fig. 7c), suggesting the function of this binding sequence. Then ChIP followed by qPCR was performed to confirm the interaction between FUS and XIAP promoter. It was validated that XIAP promoter was highly expressed in the precipitates of anti-FUS (Fig. 7d). According to DNA pull-down assay, circ0005276 and FUS can bind to the XIAP promoter to form a complex (Fig. 7e). Luciferase reporter assay showed that silencing either circ0005276 or FUS weakened the luciferase activity of XIAP promoter reporter (Fig. 7f, g). Moreover, we detected the effect of circ0005276 on the interaction between FUS and XIAP promoter. Result of ChIP assay indicated that silencing of circ0005276 impaired the affinity of FUS to XIAP promoter (Fig. 7h). Collectively, these results implied that circ0005276 co-functioned with FUS to regulate XIAP expression.

### XIAP rescued cell proliferation and migration suppressed by circ0005276 and FUS

**Fig. 8:**
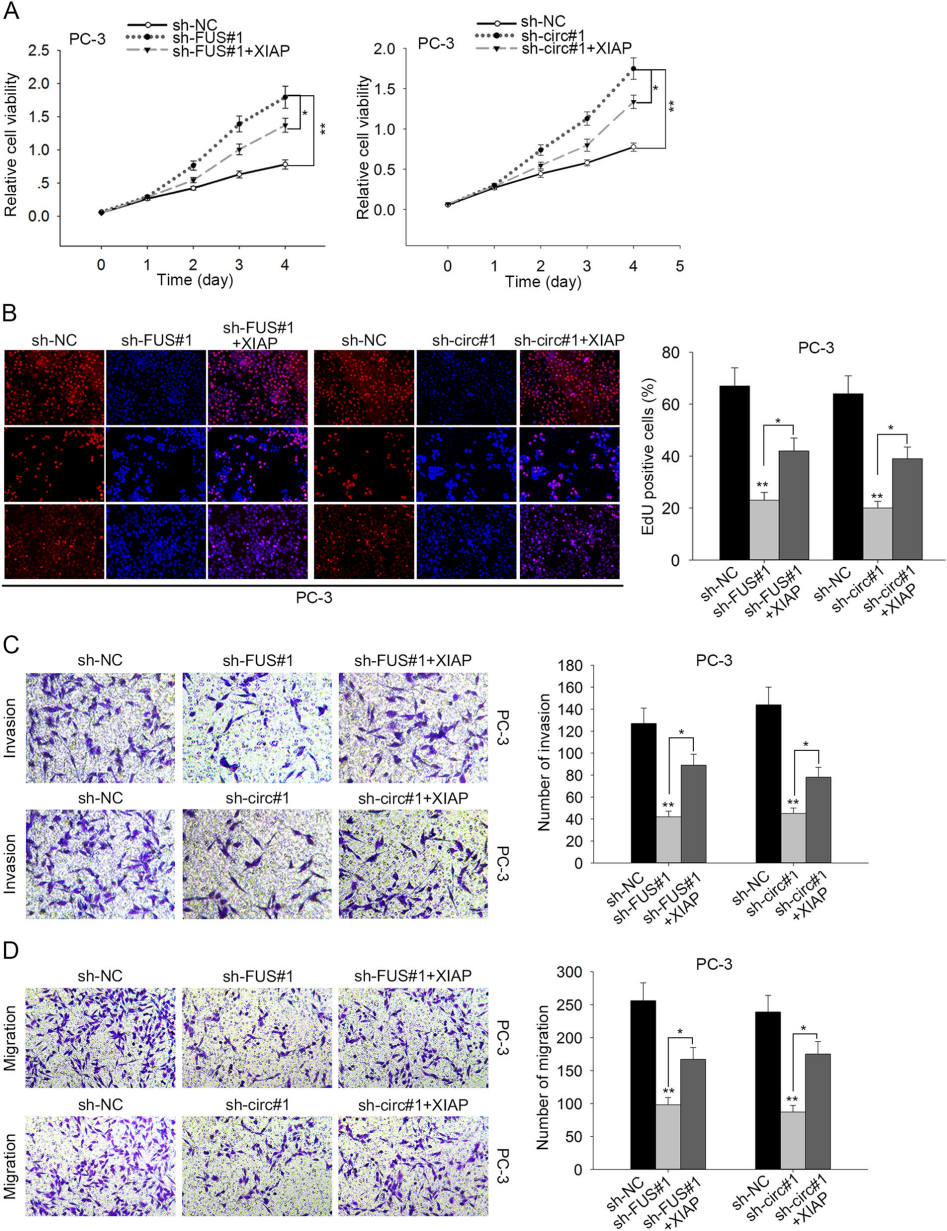
XIAP rescued cell proliferation and migration suppressed by circ0005276 and FUS. **a**–**b** Proliferation of FUS or circ0005276-downregulated PCa cells in response to the overexpression of XIAP. **c–d** Migration or invasion in indicated PCa cells. ^*^P < 0.05, ^**^P < 0.01

Next, we conducted rescue assays to demonstrate the involvement of XIAP in the PCa progression mediated by circ0005276 or FUS. CCK-8 assay and EdU revealed that overexpression of XIAP reversed the cell proliferation inhibited by the silencing of circ0005276 or FUS (Fig. 8a, b). The migratory and invasive ability were impaired by the knockdown of circ0005276 or FUS, while both migration and invasion were recovered by the introduction of XIAP (Fig. 8c, d). Altogether, results above suggested that circ0005276/FUS axis promoted cell proliferation and migration via upregulating XIAP.

## Discussion

Prostate cancer is frequent cancer which severely threatened the health of males in the world. Nowadays, more and more non-coding RNAs have been revealed to be regulators in the tumorigenesis and development of human cancers. Hence, present study aimed to identify new therapeutic targets for PCa.

In current study, XIAP was found to be a mRNA which was significantly upregulated in PCa samples. Upregulation of XIAP was further validated in PCa tissues collected from 90 patients with PCa. Notably, studies have disclosed that XIAP is closely related to the initiation and progression of malignant tumors^34,35^. Thus, we conducted further experiments to demonstrate the role of XIAP in PCa. Searching from UCSC, we found that circ0005276 is a circular RNA stem from XIAP. To analyze the role of circ0005276 in PCa, we measured its expression level at first. In consistent with XIAP, circ0005276 was also expressed higher in PCa tissues. In the present study, we silenced both XIAP and circ0005276 in PCa cell lines. And we found the positive regulation of circ0005276 on XIAP, but there was no significant effect of XIAP knockdown on the circ0005276 expression. It has been demonstrated previously, circRNAs can regulate tumor progression by acting as oncogenes or tumor suppressors^36–39^. Considering the relative high expression of XIAP and circ0005276 in PCa tissues with advanced tumor stage and metastasis, we hypothesized that they might be two oncogenes in PCa progression. Functionally, downregulation of XIAP or circ0005276 led to the depletion of cell proliferation, invasion and migration. Moreover, the EMT process was reversed in XIAP or circ0005276-downregulated PCa cell lines.

In mechanism, circRNAs can exert function in human cancers by interacting with RBPs^25–27,31^. According to the cytoplasmic and nuclear localization of circ0005276, we further applied mass spectrometry and pull-down assay to screen out a RBP which can interact with circ0005276. Combining with further mechanism experiments, we determined the interaction between circ0005276 and FUS. The expression association between circ0005276 and FUS was demonstrated to be positive. As previously reported, FUS can promote cell proliferation and migration in malignant tumors^40,41^. Similarly, we explored the function of FUS in two PCa cells through loss-of function assays. According to the experimental results, we concluded that FUS might be an oncogene in PCa by promoting cell proliferation and migration.

It has been reported that FUS can transcriptionally activate genes^42^. In the current study, we identified that FUS and circ0005276 positively regulate XIAP in PCa cell lines. Based on all above data, we hypothesized that circ0005276 might interact with FUS to regulate the transcription of XIAP in PCa. Accordingly, the effect of FUS on the XIAP transcription was analyzed. The putative binding sites between FUS and XIAP promoter were analyzed by using bioinformatics tools. Mechanism experiments demonstrated the binding of FUS to XIAP promoter. Combining with the positive regulation of FUS on XIAP expression, we confirmed that FUS transcriptionally activated XIAP. Finally, rescue assays demonstrated that the reversal effect of XIAP on the decreased cell proliferation and migration mediated by the knockdown of FUS and circ0005276. Together, our study firstly disclosed a novel circRNA0005276 as a potential therapeutic marker in PCa by revealing that circ0005276/FUS promoted the tumorigenesis and development of PCa through regulating XIAP expression.

## Supplementary information

Supplementary figure legends

Supplementary Figure 1

Supplementary Figure 2

Supplementary Figure 3

Supplementary Figure 4

Supplementary Table 1

## References

[1] Xu S (2016). Long non-coding RNA ATB promotes growth and epithelial-mesenchymal transition and predicts poor prognosis in human prostate carcinoma. Oncol. Rep..

[2] Siegel RL, Miller KD, Jemal A (2016). Cancer statistics, 2016. CA: a cancer J. clinicians.

[3] Wu J (2017). Long noncoding RNA LINC01296 is associated with poor prognosis in prostate cancer and promotes cancer-cell proliferation and metastasis. OncoTargets Ther..

[4] Chen LL (2016). The biogenesis and emerging roles of circular RNAs. Nat. Rev. Mol. Cell Biol..

[5] Kolakofsky D (1976). Isolation and characterization of Sendai virus DI-RNAs. Cell.

[6] Shang Q, Yang Z, Jia R, Ge S (2019). The novel roles of circRNAs in human cancer. Mol. cancer.

[7] Panda, A. C. & Gorospe, M. Detection and Analysis of Circular RNAs by RT-PCR. *Bio Protoc* **8**, 10.21769/BioProtoc.2775 (2018). 10.21769/BioProtoc.2775 PMC5891140 29644261

[8] Sanger HL, Klotz G, Riesner D, Gross HJ, Kleinschmidt AK (1976). Viroids are single-stranded covalently closed circular RNA molecules existing as highly base-paired rod-like structures. Proc. Natl Acad. Sci. USA.

[9] Guo JU, Agarwal V, Guo H, Bartel DP (2014). Expanded identification and characterization of mammalian circular RNAs. Genome Biol..

[10] Wang KS (1986). Structure, sequence and expression of the hepatitis delta (delta) viral genome. Nature.

[11] Chen W, Schuman E (2016). Circular RNAs in brain and other tissues: a functional enigma. Trends Neurosci..

[12] Salzman J, Gawad C, Wang PL, Lacayo N, Brown PO (2012). Circular RNAs are the predominant transcript isoform from hundreds of human genes in diverse cell types. PLoS ONE.

[13] Petkovic S, Muller S (2015). RNA circularization strategies in vivo and in vitro. Nucleic acids Res..

[14] Memczak S (2013). Circular RNAs are a large class of animal RNAs with regulatory potency. Nature.

[15] Hansen TB (2013). Natural RNA circles function as efficient microRNA sponges. Nature.

[16] Wang J (2019). Circular RNAs: a rising star in respiratory diseases. Respiratory Res..

[17] Hansen TB, Kjems J, Damgaard CK (2013). Circular RNA and miR-7 in cancer. Cancer Res..

[18] Wu J (2019). Emerging epigenetic regulation of circular RNAs in human cancer. Mol. Ther. Nucleic acids.

[19] Bach DH, Lee SK, Sood AK (2019). Circular RNAs in cancer. Mol. Ther. Nucleic acids.

[20] Yang L, Yang F, Zhao H, Wang M, Zhang Y (2019). Circular RNA circCHFR facilitates the proliferation and migration of vascular smooth muscle via miR-370/FOXO1/Cyclin D1 pathway. Mol. Ther. Nucleic acids.

[21] Huang Xing, Wang Xiao-nan, Yuan Xiao-dong, Wu Wen-yong, Lobie Peter E., Wu Zhengsheng (2018). XIAP facilitates breast and colon carcinoma growth via promotion of p62 depletion through ubiquitination-dependent proteasomal degradation. Oncogene.

[22] Xu J (2018). Overexpression of the Kininogen-1 inhibits proliferation and induces apoptosis of glioma cells. J. Exp. Clin. cancer Res.: CR.

[23] Evans MK (2018). XIAP Regulation by MNK Links MAPK and NFkappaB signaling to determine an aggressive breast cancer phenotype. Cancer Res..

[24] Yu Y (2018). XIAP overexpression promotes bladder cancer invasion in vitro and lung metastasis in vivo via enhancing nucleolin-mediated Rho-GDIbeta mRNA stability. Int. J. cancer.

[25] Wang J (2015). Regulatory roles of non-coding RNAs in colorectal cancer. Int. J. Mol. Sci..

[26] Du WW (2017). Induction of tumor apoptosis through a circular RNA enhancing Foxo3 activity. Cell death Differ..

[27] Abdelmohsen K (2017). Identification of HuR target circular RNAs uncovers suppression of PABPN1 translation by CircPABPN1. RNA Biol..

[28] Hata A (2000). OAZ uses distinct DNA- and protein-binding zinc fingers in separate BMP-Smad and Olf signaling pathways. Cell.

[29] Du WW (2017). Identifying and characterizing circRNA-protein interaction. Theranostics.

[30] Hentze MW, Preiss T (2013). Circular RNAs: splicing’s enigma variations. EMBO J..

[31] Sun H-D (2018). Down-regulation of circPVRL3 promotes the proliferation and migration of gastric cancer cells. Sci. Rep..

[32] Ishigaki S (2012). Position-dependent FUS-RNA interactions regulate alternative splicing events and transcriptions. Sci. Rep..

[33] Colombrita C (2012). TDP-43 and FUS RNA-binding proteins bind distinct sets of cytoplasmic messenger RNAs and differently regulate their post-transcriptional fate in motoneuron-like cells. J. Biol. Chem..

[34] Huang X, Wu Z, Mei Y, Wu M (2013). XIAP inhibits autophagy via XIAP-Mdm2-p53 signalling. Embo j..

[35] Carter BZ (2010). Simultaneous activation of p53 and inhibition of XIAP enhance the activation of apoptosis signaling pathways in AML. Blood.

[36] Wang Renjie, Zhang Sai, Chen Xuyi, Li Nan, Li Jianwei, Jia Ruichao, Pan Yuanqing, Liang Haiqian (2018). CircNT5E Acts as a Sponge of miR-422a to Promote Glioblastoma Tumorigenesis. Cancer Research.

[37] Yang Feng, Fang Erhu, Mei Hong, Chen Yajun, Li Huanhuan, Li Dan, Song Huajie, Wang Jianqun, Hong Mei, Xiao Wenjing, Wang Xiaojing, Huang Kai, Zheng Liduan, Tong Qiangsong (2018). Cis-Acting circ-CTNNB1 Promotes β-Catenin Signaling and Cancer Progression via DDX3-Mediated Transactivation of YY1. Cancer Research.

[38] Chen Xin, Chen Ri-Xin, Wei Wen-Su, Li Yong-Hong, Feng Zi-Hao, Tan Lei, Chen Jie-Wei, Yuan Gang-Jun, Chen Si-Liang, Guo Sheng-Jie, Xiao Kang-Hua, Liu Zhuo-Wei, Luo Jun-Hang, Zhou Fang-Jian, Xie Dan (2018). PRMT5 Circular RNA Promotes Metastasis of Urothelial Carcinoma of the Bladder through Sponging miR-30c to Induce Epithelial–Mesenchymal Transition. Clinical Cancer Research.

[39] Chen B (2018). circEPSTI1 as a prognostic marker and mediator of triple-negative breast cancer progression. Theranostics.

[40] Ward CL (2014). A loss of FUS/TLS function leads to impaired cellular proliferation. Cell death Dis..

[41] Deng Jianwen, Wang Peng, Chen Xiaoping, Cheng Haipeng, Liu Jianghong, Fushimi Kazuo, Zhu Li, Wu Jane Y. (2018). FUS interacts with ATP synthase beta subunit and induces mitochondrial unfolded protein response in cellular and animal models. Proceedings of the National Academy of Sciences.

[42] Tan AY, Riley TR, Coady T, Bussemaker HJ, Manley JL (2012). TLS/FUS (translocated in liposarcoma/fused in sarcoma) regulates target gene transcription via single-stranded DNA response elements. Proc. Natl Acad. Sci. USA.

