# Supplementary material for: Circular RNA circ0005276 promotes the proliferation and migration of prostate cancer cells by interacting with FUS to transcriptionally activate XIAP
Source: Cell Death Dis. 2019 Oct 17;10(11):792. doi: 10.1038/s41419-019-2028-9 (PMC6797747; doi:10.1038/s41419-019-2028-9)
Supplement: Supplementary file 1 — Supplementary figure legends [file 41419_2019_2028_MOESM1_ESM.docx]

**Supplementary figure legends**

**Supplementary Figure 1.** (A) Microarray analysis of dysregulated mRNAs in PCa samples. (B) GO analysis of upregulated mRNAs. (C) Enriched pathways were analyzed by KEGG analysis.

**Supplementary Figure 2.** RNA sequencing for circ0005276.

**Supplementary Figure 3.** (A-B) Knockdown of XIAP or circ0005276 by specific shRNAs. (C) Quantification of protein levels in Figure 4C. (D) Tumors derived from cells transfected with sh-NC, sh-circ0005276 or sh-XIAP were collected and observed. (E-F) Tumor volume and weight in three groups were calculated. ^*^P < 0.05, ^**^P < 0.01.

**Supplementary Figure 4.** (A) Quantification of protein levels in Figure 6F-G. (B-D) Cell proliferation, invasion and migration were separately examined by EdU and transwell assays. ^**^P < 0.01.
