# Supplementary figures and images for: Circular RNA circ0005276 promotes the proliferation and migration of prostate cancer cells by interacting with FUS to transcriptionally activate XIAP
Source: Cell Death Dis. 2019 Oct 17;10(11):792. doi: 10.1038/s41419-019-2028-9 (PMC6797747; doi:10.1038/s41419-019-2028-9)

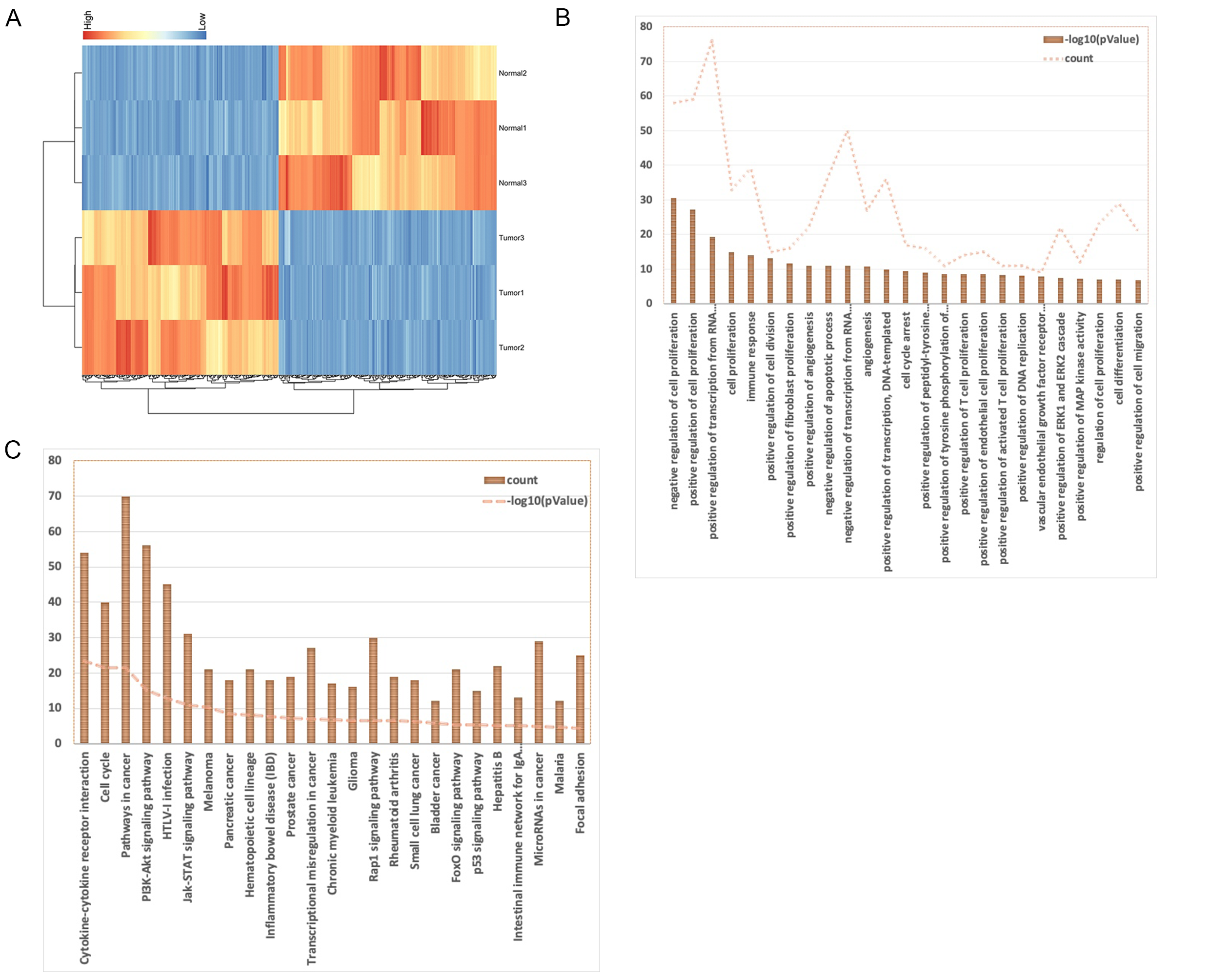

Supplement: Supplementary file 2 — Supplementary Figure 1 [file 41419_2019_2028_MOESM2_ESM.tif]

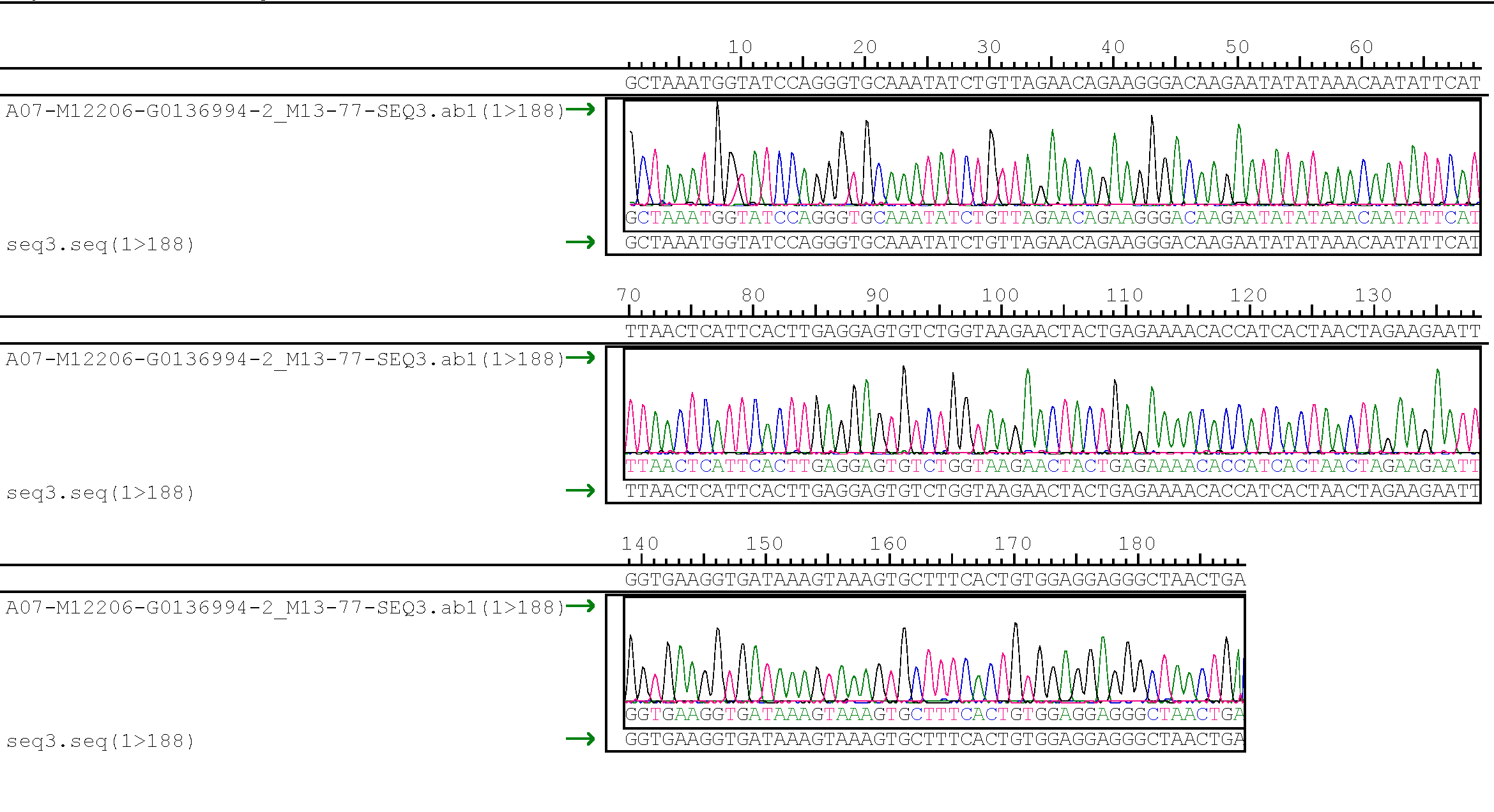

Supplement: Supplementary file 3 — Supplementary Figure 2 [file 41419_2019_2028_MOESM3_ESM.tif]

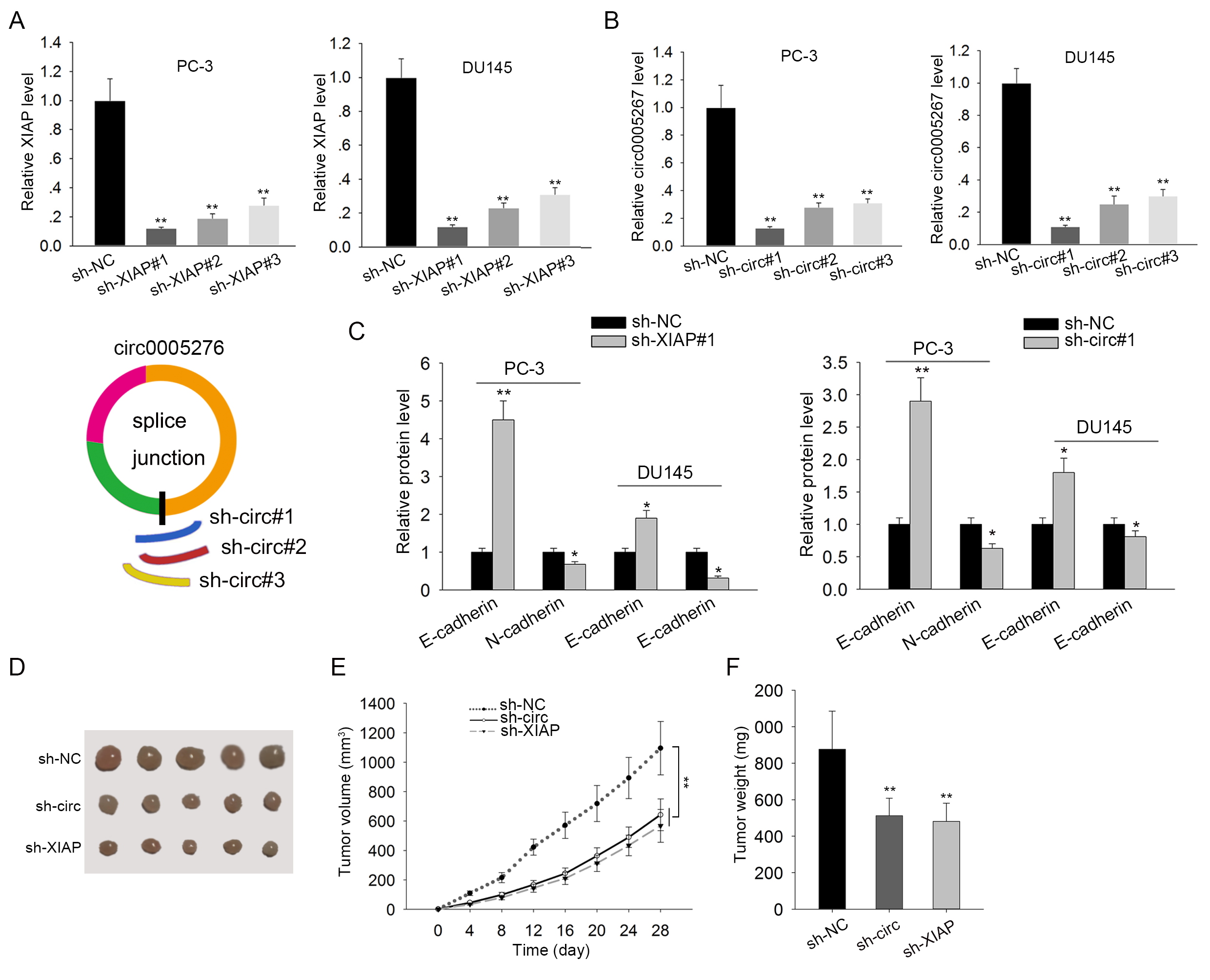

Supplement: Supplementary file 4 — Supplementary Figure 3 [file 41419_2019_2028_MOESM4_ESM.tif]

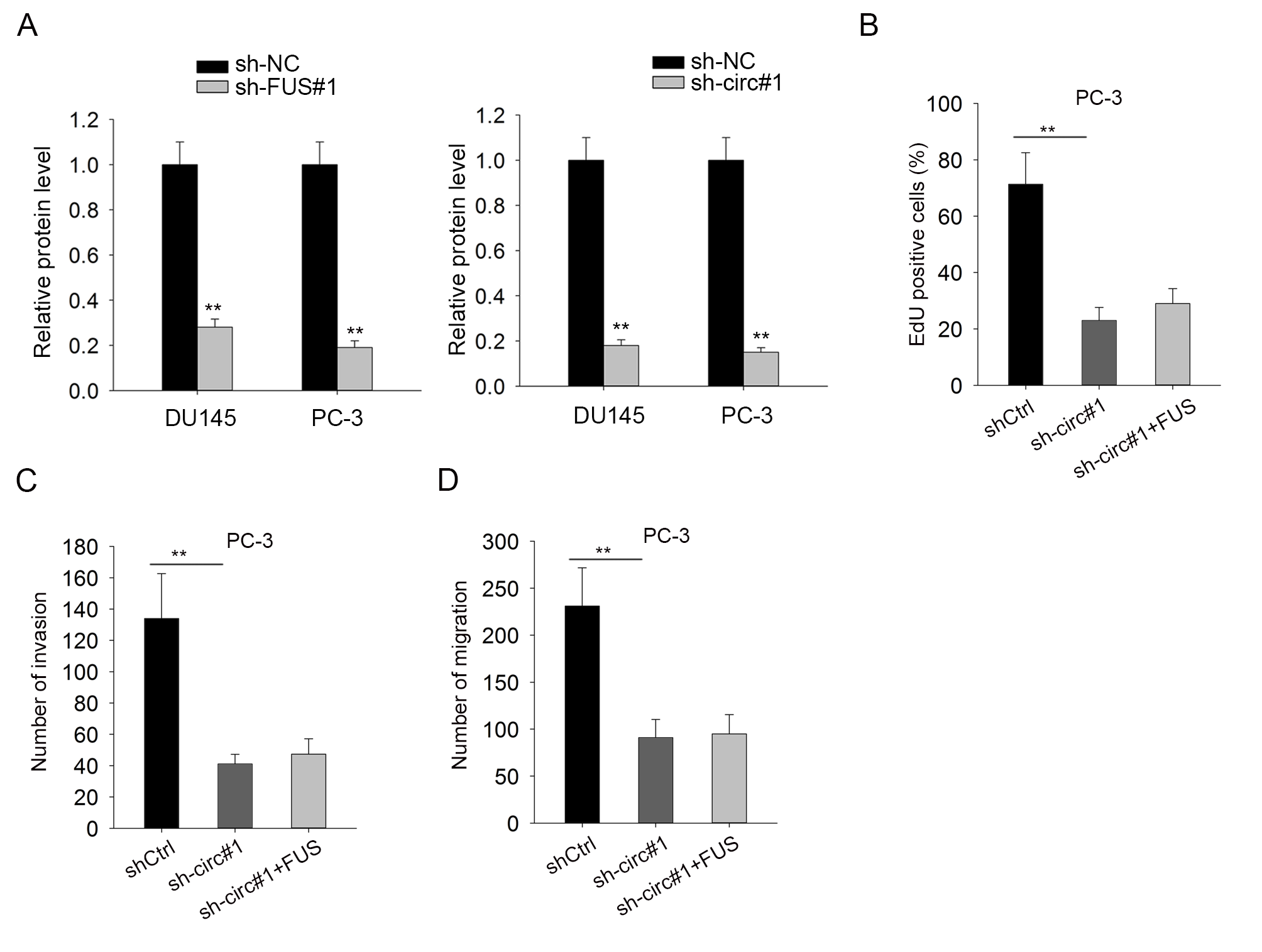

Supplement: Supplementary file 5 — Supplementary Figure 4 [file 41419_2019_2028_MOESM5_ESM.tif]
